# Spatial Distribution of Intracellular Ion Concentrations in Aggregate‐Forming HeLa Cells Analyzed by μ‐XRF Imaging

**DOI:** 10.1002/open.202200024

**Published:** 2022-04-01

**Authors:** Andreas Gräfenstein, Christoph Rumancev, Roland Pollak, Benjamin Hämisch, Vanessa Galbierz, Walter H. Schroeder, Jan Garrevoet, Gerald Falkenberg, Tobias Vöpel, Klaus Huber, Simon Ebbinghaus, Axel Rosenhahn

**Affiliations:** ^1^ Analytical Chemistry – Biointerfaces Ruhr University Bochum 44801 Bochum Germany; ^2^ Institute of Physical and Theoretical Chemistry TU Braunschweig Rebenring 56 38106 Braunschweig Germany; ^3^ Physical Chemistry University of Paderborn 33098 Paderborn Germany; ^4^ Deutsches Elektronen-Synchrotron DESY Notkestrasse 85 Hamburg Germany; ^5^ Nanotech Consulting Liblarer Strasse 8 50321 Brühl Germany; ^6^ Physical Chemistry II Ruhr University Bochum 44801 Bochum Germany

**Keywords:** huntingtin, potassium, pseudoisocyanine chloride, X-ray fluorescence, zinc

## Abstract

Protein aggregation is a hallmark of several severe neurodegenerative disorders such as Huntington's, Parkinson's, or Alzheimer's disease. Metal ions play a profound role in protein aggregation and altered metal‐ion homeostasis is associated with disease progression. Here we utilize μ‐X‐ray fluorescence imaging in combination with rapid freezing to resolve the elemental distribution of phosphorus, sulfur, potassium, and zinc in huntingtin exon‐1‐mYFP expressing HeLa cells. Using quantitative XRF analysis, we find a threefold increase in zinc and a 10‐fold enrichment of potassium that can be attributed to cellular stress response. While the averaged intracellular ion areal masses are significantly different in aggregate‐containing cells, a local intracellular analysis shows no different ion content at the location of intracellular inclusion bodies. The results are compared to corresponding experiments on HeLa cells forming pseudoisocyanine chloride aggregates. As those show similar results, changes in ion concentrations are not exclusively linked to huntingtin exon‐1 amyloid formation.

## Graphical abstract

**Quantitative μ‐X‐ray fluorescence imaging** was used in combination with rapid freezing to study the elemental distribution in huntingtin‐expressing HeLa cells. A threefold increase in zinc and a 10‐fold enrichment of potassium was found. Corresponding experiments on HeLa cells forming pseudoisocyanine chloride aggregates showed similar results. Both these effects can be attributed to a cellular stress response.

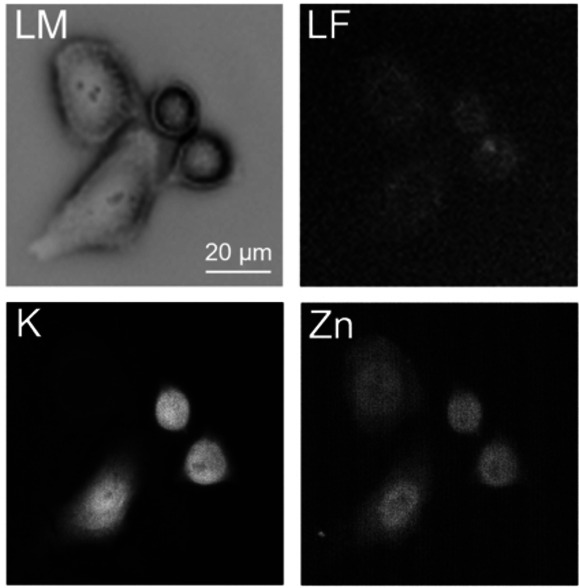

## Introduction

Metal ions such as alkaline and earth alkaline metal ions or first row transition metal ions play a profound role in cell physiology and are also associated with neuropathological processes. Na^+^ and K^+^ ions are involved in cell volume regulation, apoptosis or neuronal communication, and closing and opening of the respective channels elicit the action potential in neurons.^[1]^ Cell volume regulation under osmotic stress is controlled by efflux of K^+^ from the cell, while shrinkage is controlled by Na^+^ influx.^[2]^ An analysis of the brain sections of patients with Lewy bodies and known dementia show a disturbed Na^+^/K^+^ equilibrium.^[3]^ Apoptotic processes in a wide range of cell types and conditions are commonly associated with K^+^ efflux and Na^+^ influx.^[4]^ Increased Na^+^ and K^+^ levels were found in the brain tissue of Alzheimer's disease patients which was attributed to a decreased level of Na^+^/K^+^ ATPase and Na^+^‐dependent glutamate transporters.^[5]^

First row transition metal ions such as zinc (Zn), copper (Cu), manganese (Mn) or molybdenum (Mo) are essential in cell physiology due to their central role in enzymatic catalysis, as shown by, for example, the occurrence of Cu^2+^ and Zn^2+^ in superoxide dismutase (SOD). SOD plays a pivotal role in the maintenance of reactive oxygen species (ROS) that emerge from oxidative processes, for example, due to cellular signaling or oxidative stress. However, under cellular stress conditions, thiol oxidation can lead to Zn^2+^ release to the cytoplasm, triggering further ROS generation and inactivation of the enzyme in a feedback loop,^[6]^ leading to Zn‐mediated cellular toxicity. Cell injury by ROS can, in turn, lead to the damage of potassium channels.^[7]^ Zn^2+^ and Cu^2+^ also play a crucial role in the formation of amyloid β (Aβ) plaques in Alzheimer's disease where they are found to be enriched.^[8]^ However, this effect is specific to the Aβ since the protein contains histidine metal binding sites.^[9]^

Classical measurements of ion concentrations and permeability of cellular membranes rely on a wide range of conductive methods in electrophysiology.^[1]^ These methods are complemented by specialized applications of absorption/emission spectroscopy or mass spectrometry as well as by molecular sensors for colorimetric analysis.^[10]^ However, these techniques are often disruptive to cells, lack spatial resolution, or require the delivery of sensors that are commonly difficult to calibrate in intracellular environments.

Synchrotron‐based Nano‐ and Microprobe XRF Imaging enables the simultaneous acquisition of elements and their distribution with spatial resolutions well below the micrometer regime. Owing to its high sensitivity and penetration depth, XRF allows to quantitatively analyze trace element distributions in biological samples, for example, cells or tissue sections.[^11^, ^12^] It was applied to study a multitude of biological systems, including the elemental distribution in plants,^[13]^ melanosomes,^[14]^ juvenile barnacles,^[15]^ and damselfly wings.^[16]^ In particular, the distribution of biologically relevant elements was investigated for different cells, for example human neutrophils,^[17]^ mouse embryonic fibroblast cells,^[18]^ PC12 cells,^[19]^ human cancer cells after treatment with drugs,[^20^, ^21^, ^22^] and Alzheimer‐affected brain cells.[^8^, ^23^, ^24^] Despite these applications of XRF for the assessment of Aβ subcellular localization and its amyloid‐specific ion enrichment, the XRF technique was so far, to the best of our knowledge, not used to resolve elemental distributions in other amyloid‐associated pathologies.

To preserve the native ion and element distributions during the measurement, a suitable preparation protocol is critical. A suitable method, which keeps the cells closest to their natural hydrated state,^[25]^ is shock‐freezing the sample using a high‐cooling rate, thus locking all ions in their current position while circumventing the formation of ice‐crystals.[^26^, ^27^] This can be achieved by plunge freezing, that is, rapid immersion in liquids with high cooling‐rates.[^28^, ^29^] The procedure avoids the need for chemical fixation and drying, which can induce changes to the spatial organization and elemental distribution.^[30]^ In addition to preserving the morphology, radiation damage is strongly reduced.^[31]^

We recently described a new cryogenic sample environment at the Hard X‐ray Micro/Nano‐Probe beamline P06 at PETRA III, DESY, which utilizes a large solid detection angle of up to 1.1 sr, enabling high count rates of up to 10^6^ counts per second, which in turn enables short acquisition times, while also maintaining high sensitivity and good energy resolution. Furthermore, as a cryogenic vacuum chamber, it functions as a sample environment with a cooled and isolated sample stage and base pressures in the UHV regime to avoid contamination of the cryogenically preserved samples.[^29^, ^32^]

Using this technique, we studied the change of P, S, K, and Zn areal masses in the neuropathological process of aberrant protein aggregation. As a model system we chose HeLa cells that overexpress huntingtin protein exon 1 (Htt‐ex1). HTT is associated with the onset of Huntington's disease (HD), a severe neurogenerative disease caused by a polyglutamine expansion in Htt‐ex1.^[33]^

We compared a pathogenic expanded protein (Q72) that forms intracellular aggregates to native protein (Q25) that remains soluble. The question was pursued if in these two in‐cell aggregation model systems Zn levels are affected and if a colocalization with inclusion bodies (IBs) – aggregates of misfolded proteins closely associated with HD – occurs. As the cryogenic preparation allows the quantification of the intracellular K^+^ levels, we also studied how the presence of Q25/Q72 affects the K^+^ equilibrium. To test if the observed changes in ion levels are specific to the overexpression and aggregation of the Htt‐ex1 protein, we used comparative experiments studying a small molecule dye, pseudoisocyanine chloride (PIC), that forms intracellular J‐aggregated structures.^[34]^ These aggregates show an intense, characteristic and sharp absorption and fluorescence band at around 573 nm, leading to highly sensitive detection of aggregation inside cellular environments by fluorescence microscopy.

## Results and Discussion

**Figure 1:**
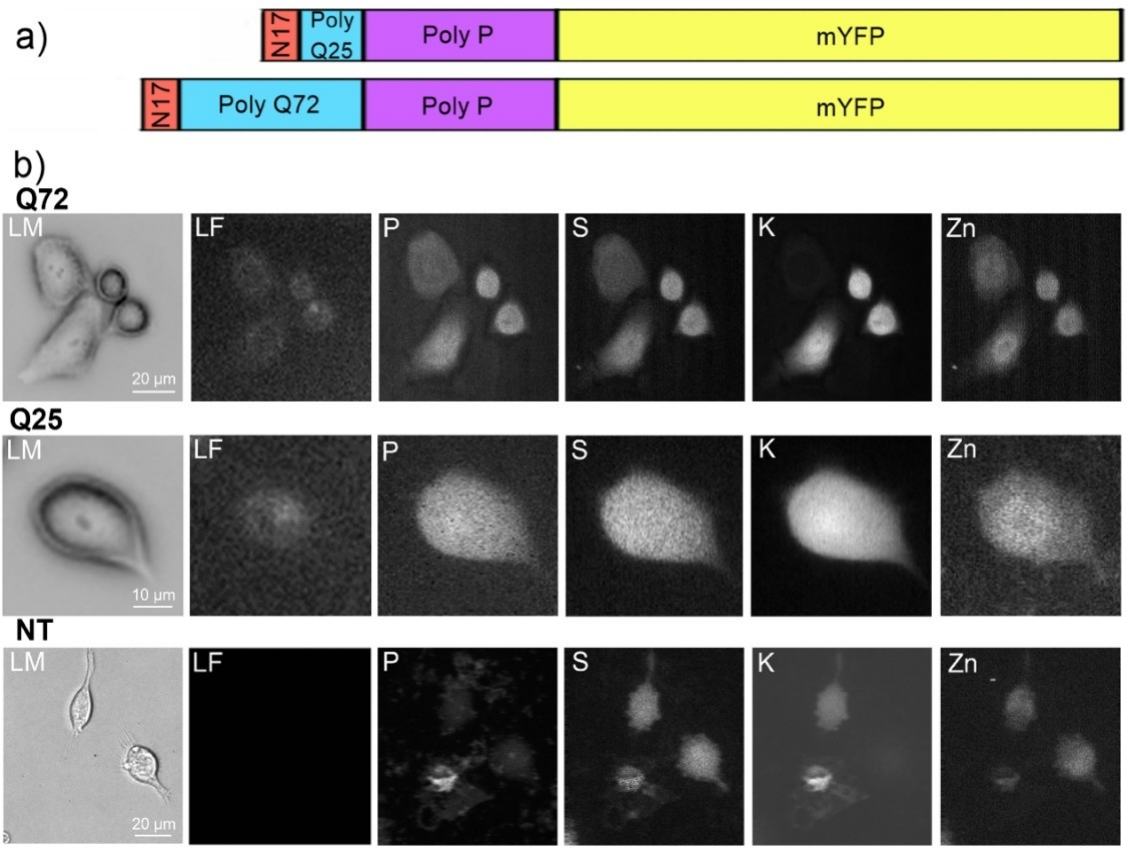
a) Domain structure of the overexpressed Htt‐ex1‐mYFP protein. An N‐terminal domain (N17) and a polyproline domain (PolyP) flank the PolyQ domain that is native (Q25) or extended (Q72). mYFP is used for C‐terminal fluorescence detection; b) exemplary phase contrast microscopy (LM) and visible‐light fluorescence (LF) microscopy as well as X‐ray fluorescence images of individual HeLa cells. Cells were transfected with Htt‐ex1 proteins with different polyglutamine lengths (Q25 & Q72) and compared to untreated, non‐transfected cells (NT).

**Figure 2:**
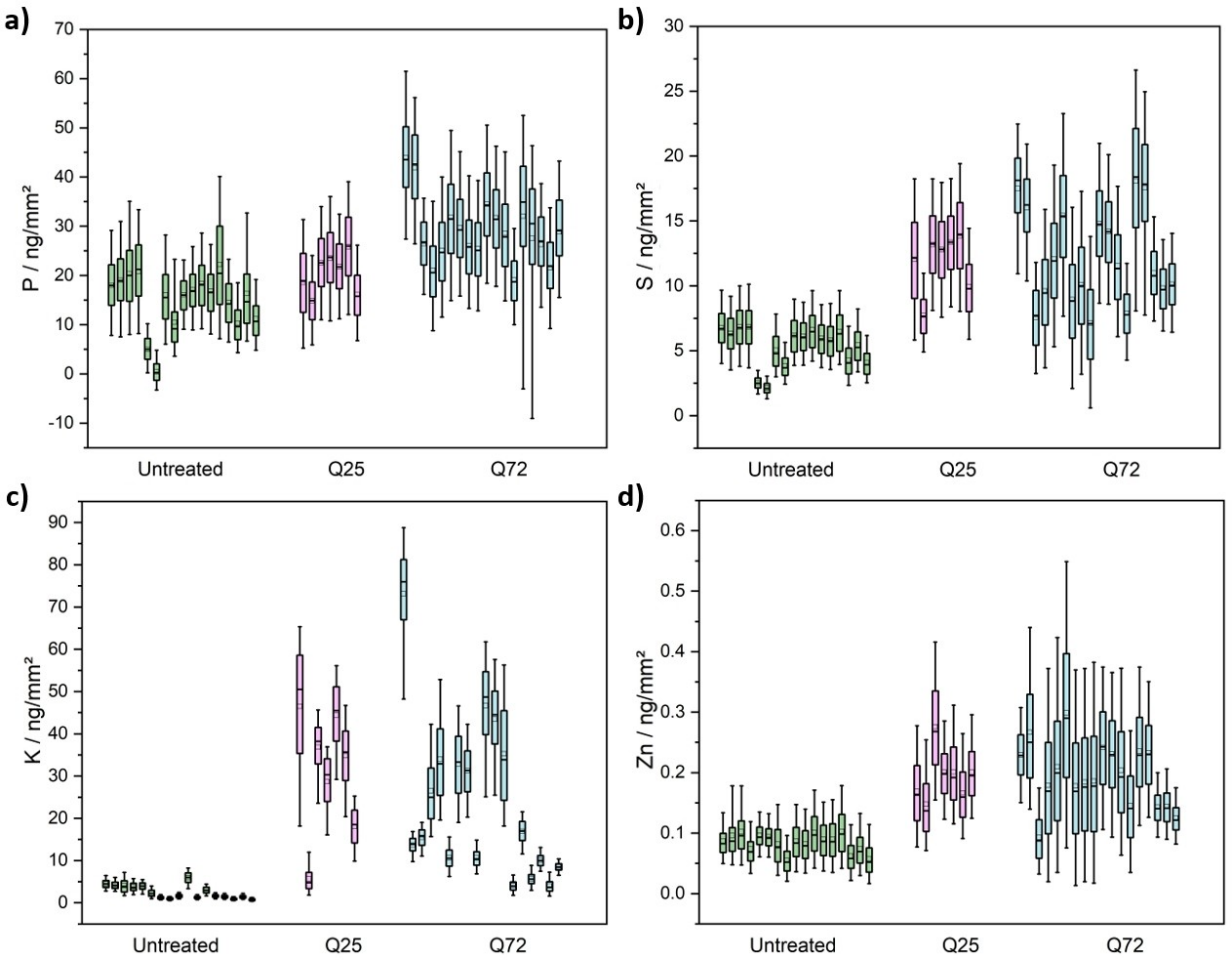
Concentrations determined within individual Htt‐transfected (Q25 & Q72) and non‐transfected HeLa cells for the elements a) phosphorus, b) sulfur, c) potassium, and d) zinc. The concentrations were determined for each pixel in the cells and the distribution is represented as a boxplot. The colored boxes represent values from 25 %–75 %, while the whiskers represent values from 5 % −95 %. A total of 171,660 XRF spectra recorded on 42 cells were analyzed.

Htt‐ex1‐transfected cells with two different polyglutamine lengths (Q25 & Q72) (Figure 1 a) and non‐transfected HeLa cells as a reference were analyzed by phase contrast (LM), visible‐light fluorescence (LF) and nanoprobe X‐ray fluorescence microscopy (Figure 1b, Figure S3). The shape of the cells in the XRF images of Zn, P, S, and K correspond well with the morphology of the cells seen in the corresponding visible‐light micrographs. The gray‐scale intensity represents the measured area concentrations of the elements (Zn, P, S, and K). Due to the decreasing thickness of the cells towards their periphery and the fact that the XRF images show a projection along the beam axis, a decrease of the XRF signal towards the cellular periphery can be observed for all detected elements. Figure 2 shows the population analysis for all cells measured, whereas each box in the boxplot represents the area concentrations analyzed throughout the area of one individual cell. For the untreated cells, 17 individual cells, for the Q25 cells, 7 cells, and for the Q72 cells, 18 cells and a total of >170,000 spectra were analyzed.

Across the entire analyzed dataset, the P, S, K, and Zn values are statistically significantly lower in the untreated cells than in the Q72‐expressing cells (p<0.05) (Table S1). K levels were increased from 2.5 to 22.9 ng mm^−2^, S levels from 5.4 to 12.2 ng mm^−2^, P levels from 14.9 to 29 ng mm^−2^, and Zn values from 0.08 to 0.2 ng mm^−2^ (Table 1). For the Q25 treatment, only the S, K, and Zn levels are statistically significantly higher, while the P levels are not significantly affected (p>0.05) (Table S1). In turn, between Q25 and Q72, the P values are statistically significantly different (p<0.05), which is not the case for all other elements (p>0.05) (Table S1). In relative numbers, K levels were substantially enhanced by a factor of 12 in Q25 and by a factor of 9 in Q72. P levels in the transfected cells were enhanced by a factor of 1.4 in Q25‐ and 1.9× in Q72‐transfected cells. S was enhanced by a factor of 2.2 in Q25 and by a factor of 2.3 in Q72‐transfected cells. Zn concentrations of the different cells show a slight increase of 2.4× (Q25) and 2.5× (Q72) in the case of the transfected cells.

**Table 1:** Average area concentrations (areal masses) and standard deviations of the HeLa cells investigated in Figure 1.^[a]^

	P [ng mm^−2^]	S [ng mm^−2^]	K [ng mm^−2^]	Zn [ng mm^−2^]
Untreated	14.87±5.77	5.37±1.49	2.53±1.58	0.084±0.015
Q25	20.44±4.03	11.84±2.22	30.67±14.62	0.194±0.042
Q72	28.95±6.46	12.18±3.64	22.91±18.27	0.196±0.053

[a] In total, 25 huntingtin‐transfected cells (Q25: 7, Q72: 18), and 17 untreated, non‐transfected cells have been investigated.

In addition to the differences in the average intracellular area concentrations, we noted a strong heterogeneity between different Htt‐ex1‐expressing cells. While the potassium levels in the non‐transfected cells showed a rather narrow distribution for the different cells, the potassium distribution in the Q25‐ and Q72‐transfected cells exhibited strong variations. We also noted that the XRF images of P, S, and Zn show a much smaller variation between different cells. The magnitude of this difference becomes apparent when comparing the standard deviations of the wild type and the Q72 cells (Table 1). While for P only a very weak increase by a factor of 1.1 was observed, for S, the standard deviation increases by a factor of 2.4 and for Zn by 3.5. For K, most remarkably, a 12× increase was found.

**Figure 3:**
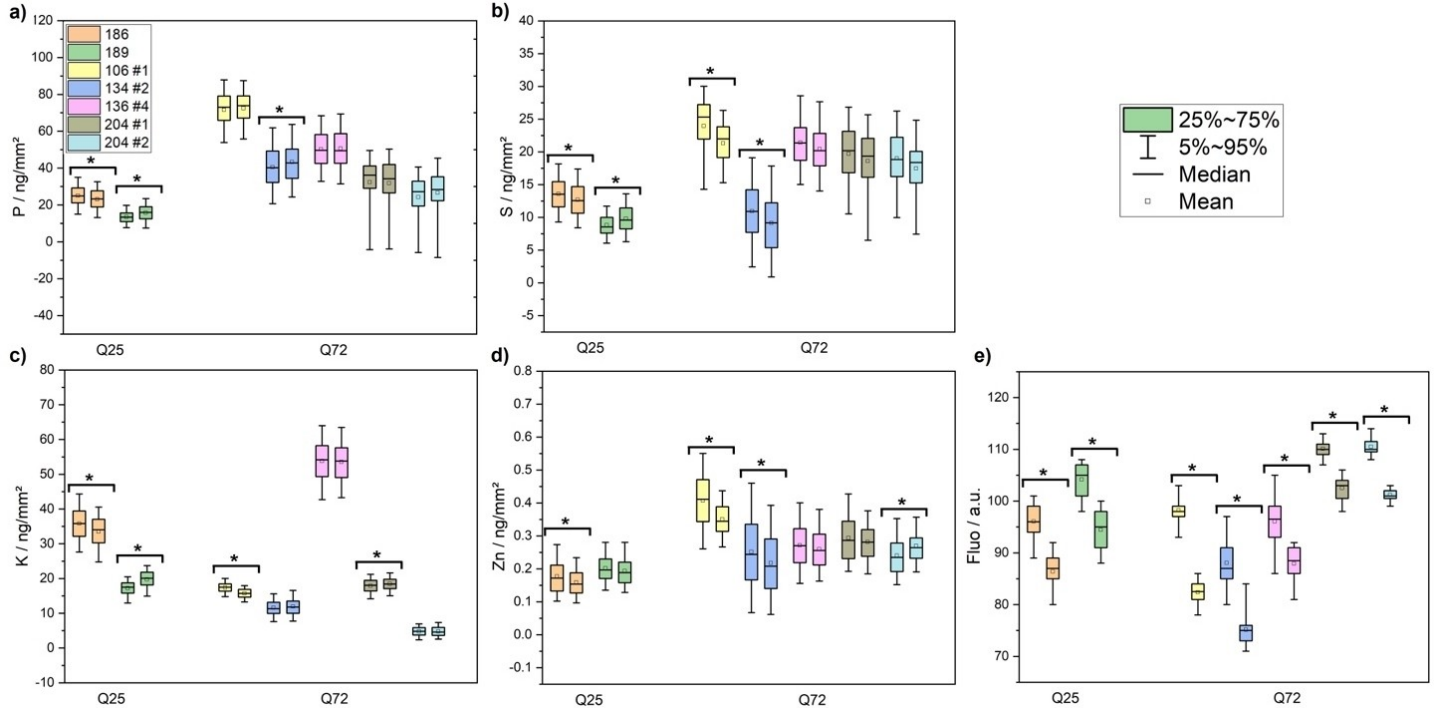
Comparison of local intracellular area concentrations of IB‐containing Htt‐ex1 cells for a) phosphorus, b) sulfur, c) potassium, and d) zinc. The left data point of the data pairs represents the concentration in areas containing IBs, the right data point represents regions with the same area but diffuse (soluble) Htt‐ex1. The position of the inclusion bodies was located by visible light fluorescence images of the cells. Each box color represents one Htt‐ex1‐transfected HeLa cell. Cells with significant differences between the two regions (p<0.05) were marked with an asterisk. The difference in mYFP fluorescence intensity for the areas within the respective individual cells is shown in e). The mYFP fluorescence intensity was significantly different for all box plot pairs (p<0.05). The corresponding p‐values are summarized in Table S2.

A comparison of the subcellular area concentration levels of P, S, K, and Zn in the cytoplasm and at the location of the inclusion bodies (IBs) of Htt‐ex1 expressing cells is shown in Figure 3. IBs were localized by light fluorescence microscopy and correlated with the corresponding XRF maps. This was achieved by determining the areal mass in a small region of interest within the cells at the localization of the IBs and a subsequent comparison to the adjacent area with diffuse (soluble) Htt‐ex1 while maintaining the area and size of the region of interest. A more detailed description and an exemplary selection procedure can be found in the Supporting Information (Figure S2). ANOVA analysis revealed that the intensity of the visible light fluorescence was statistically significantly (p<0.05) enhanced in the IB‐containing ROIs compared to the adjacent regions (Figure 3e), while for the XRF data, only in 50 % of the cases statistically significant differences (p<0.05) occurred between the two ROIs within the same cell (Figure 3a–d). In several of the statistically different cases, opposing trends, that is, increases and decreases were detected. In particular, for the K levels, where the overall levels showed a statistically significant increase between WT and transfected cells (Table 1), no noticeable difference between the IBs and the adjacent areas were found (Figure 3a–d, Table S2). Thus, the analyzed elements in the IB‐containing regions compared to the non‐IB‐containing cytoplasm were neither locally depleted nor enhanced.

**Figure 4:**
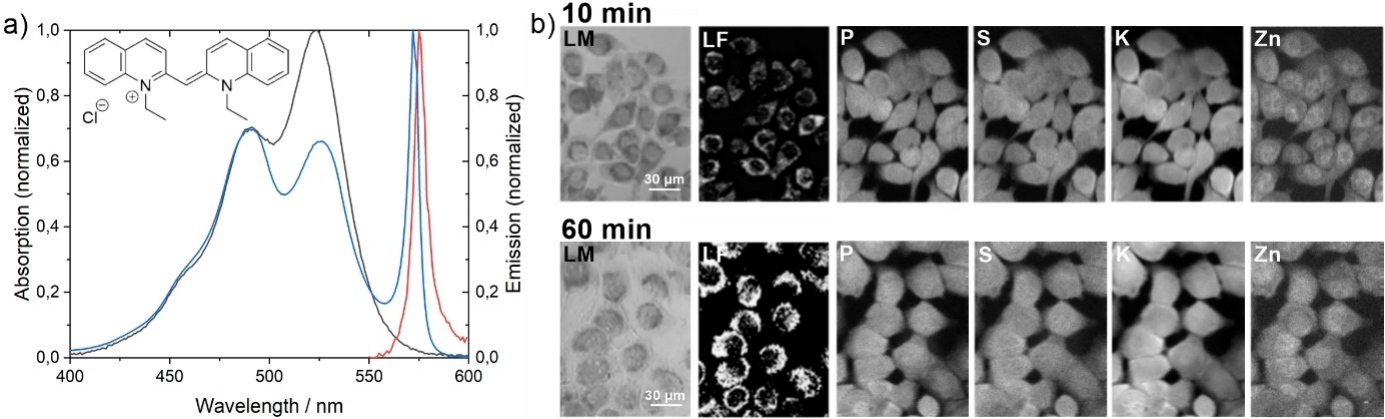
a) Normalized absorption spectrum of monomeric PIC (black) and the J‐aggregated PIC (blue) and its normalized fluorescence emission of the aggregated state (red). b) Phase contrast microscopy (LM), visible‐light fluorescence microscopy (LF) and X‐ray fluorescence images of PIC‐aggregate‐containing cells, 10 min and 60 min after treatment. Distribution of phosphorous, sulfur, potassium, and zinc after different incubation times in a PIC‐containing medium were examined.

**Figure 5:**
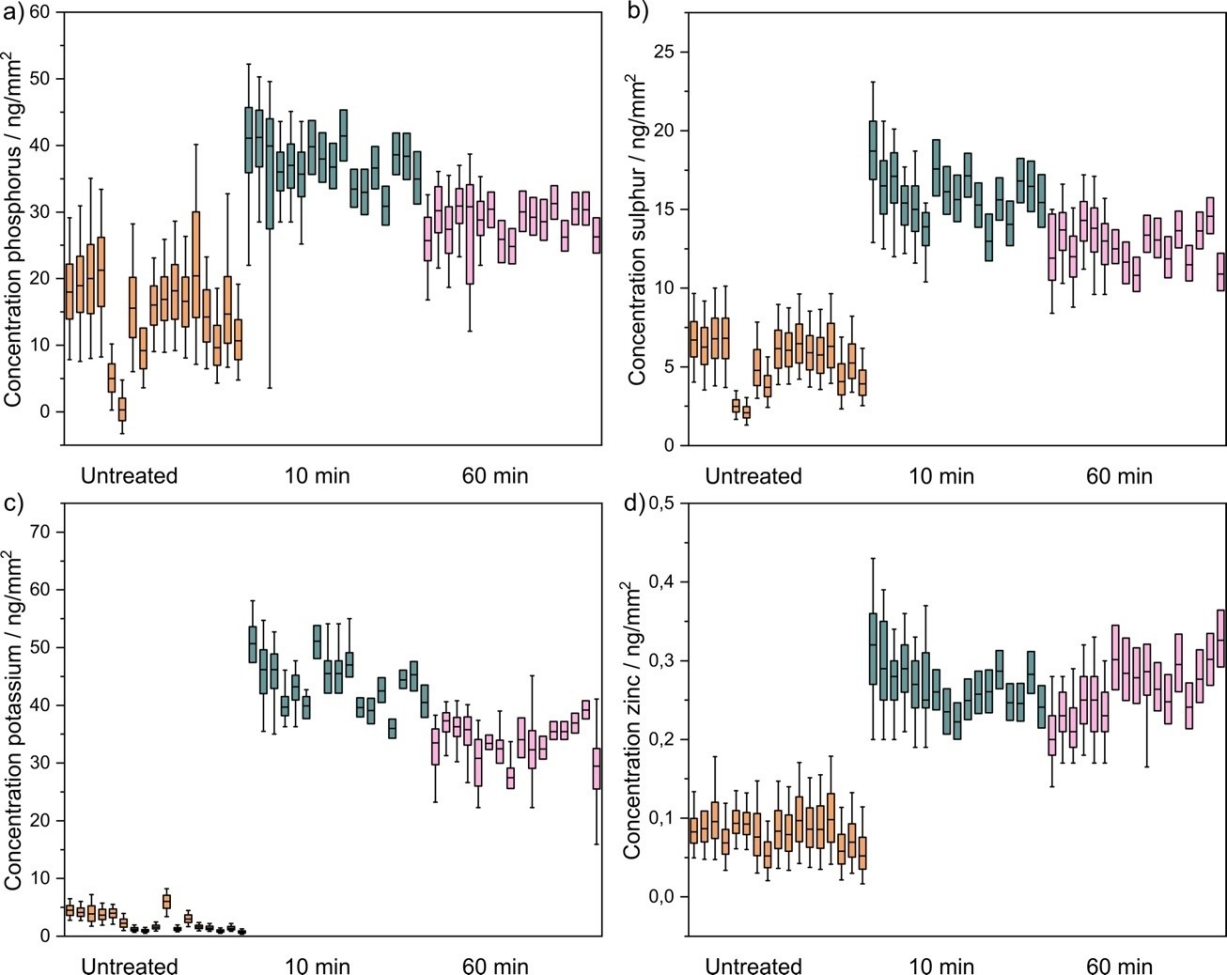
Intracellular element area concentrations in HeLa cells 10 min and 60 min after PIC treatment for the elements a) phosphorus, b) sulfur, c) potassium, and d) zinc. The concentrations were determined for each pixel in the cell and the distribution per cell is represented as a boxplot. The colored boxes represent values from 25 %–75 %, while the whiskers represent values from 5 %–95 %. A total of 303207 XRF spectra recorded on 114 cells were analyzed, only 134771 from 51 cells are shown as boxplots for the sake of clarity.

In addition to the study of Htt expressing cells, we studied pseudoisocyanine chloride (PIC) aggregates in HeLa cells to investigate whether the observed changes in element concentration were specific to the Htt‐ex1 protein. PIC is cell‐permeable and forms J‐aggregates in cells that resemble amyloid‐forming proteins and its unique emission band in the aggregated (not monomeric) state (Figure 4a) allows for fluorescence detection. The Htt‐ex1‐transfected cells were compared to PIC containing cells that were measured after 10 min and 60 min of PIC treatment (Figures 4, Figure 5, Table 2, Table S3). In agreement with the kinetic experiments in earlier studies,^[40]^ PIC aggregates were observed in the cytoplasm of the HeLa cells after 10 min with further growth in size and a 2.2‐fold increase of fluorescence intensity after 60 min. A decline in cell viability was observed 60 min after treatment, which was manifested by the typical shrinkage of cells.

**Table 2:** Average element area concentrations (areal masses) and standard deviations of HeLa cells treated with PIC.^[a]^

	P [ng mm^−2^]	S [ng mm^−2^]	K [ng mm^−2^]	Zn [ng mm^−2^]
Untreated	14.87±5.77	5.37±1.49	2.53±1.58	0.084±0.015
10 min	38.08±2.00	16.00±1.50	43.76±3.57	0.280±0.010
60 min	28.78±1.79	13.01±0.87	33.78±2.33	0.230±0.010

[a] In total, 97 PIC‐treated and 17 untreated cells were investigated.

Despite the clear enrichment of PIC aggregates in the cytoplasm, with a large contrast in LF microscopy between aggregate‐containing and non‐aggregate‐containing areas (see Figure 4, 60 min), a homogeneous distribution of P, S, K, and Zn within the cells both after 10 min and 60 min was observed in the XRF images (Figure 4b). A quantitative analysis of the average area concentration of elements across the analyzed cell populations of PIC treated cells and controls is shown in Figure 5 and Table 2. After a 10 min treatment with PIC, we observed an increase of P, S, and Zn by a factor of ≈3 and a 17‐fold increase of the concentration of K. The differences between all three treatments were statistically significant (p<0.05) in all cases (Table S3). In contrast to the Htt‐ex1 data, the standard deviation across the different cells is similar for the investigated elements, indicating a similar response of the cells to the PIC uptake.

μ‐XRF imaging of cryoprotected samples is unique in spatially resolving ion concentrations within single cells, compared to molecular sensors, that can themselves interfere with biomolecular components (e. g., associate with protein aggregates) and their response depends on the chosen probes. Using this X‐ray based technology, we were able to quantify and compare the P, S, K, and Zn levels in Htt‐ex1 transfected cells, PIC‐treated cells, and their untreated wild type controls. In addition, the local analysis of intracellular distribution of these elements allowed to separately analyze and compare the elemental area concentrations in the regions with aggregates present. Despite significantly higher levels of P, K, S, and Zn in the transfected cells, we did not find significant local accumulation of P, S, K, and Zn in Htt‐ex1 or PIC aggregates compared to the surrounding cytosol. This is still a remarkable result to report, since Htt‐ex1‐containing IBs constitute a rather distinct heterogenous subcellular environment containing a wide range of different proteins and biomolecules, for example associated with the proteasome complex or the chaperone machinery.^[41]^ Our hypothesis was that the change in environmental conditions compared to the cytoplasm could in fact lead to a heterogenous portioning of the elemental concentrations, as observed for the amyloid β, despite the absence of metal binding sites within amyloids. As such, the lack of specific ion‐binding sites in Htt‐ex1 appears to lead to Zn concentrations in regions containing aggregates that are indistinguishable from the surrounding cytosol. Similarly, the lack of S enrichment in Htt‐ex1 aggregates could be related to the fact that there are no cysteines in the sequence. A change in P could occur due to posttranslational modifications (phosphorylation of Thr‐3 and Ser‐13 and Ser‐16 in the N‐terminal domain of Htt‐ex1) that was however not detected in this study.

Also, at the intracellular locations where PIC aggregates occur, visible by the strongly enhanced contrast in the fluorescence images, the local area concentrations of the intracellular elemental levels in the XRF images remained unchanged. It is known from *in vitro* experiments in dilute and crowded (cytomimetic)^[40]^ media that PIC aggregates form densely packed fibers, which are up to 600 nm long.^[42]^ Those fibers have a cylindrical shape with six double‐stranded units whose arrangement has a diameter of 2.3 nm.^[43]^ As neither an enrichment nor a depletion was observed in areas containing PIC aggregates, our experiments suggest that smaller PIC aggregates could be homogeneously distributed along with cytoplasmic components, similar to what was observed for Htt‐ex1 aggregates. This would be in agreement with the detection of huntingtin fibrils of a length of 80–90 nm in huntingtin‐induced IBs by super‐resolution microscopy.^[44]^ The fibrils were found to be imbedded in IBs with a structure similar to the one observed in *in vitro* experiments.^[44]^ While no local enhancements at specific locations of aggregates within the cells were observed, several overall element levels within the Q25/72‐expressing and PIC‐containing cells were found to be different from the corresponding area concentrations within the untreated and non‐transfected controls.

The Zn^2+^ concentration is known to be tightly regulated in cells due to its crucial role to constitute transcription factors, enzymes, channels, or growth factors.^[45]^ Zn transporters regulate Zn^2+^ homeostasis: ZnT‐family efflux transporters reduce cytoplasmic Zn^2+^ levels by transporting Zn^2+^ out of the cell, while ZIP‐family influx transporters increase cytosolic Zn^2+^ levels by enriching Zn^2+^ in the cytosol from the extracellular fluid.^[46]^ Zn^2+^ concentration can vastly vary between cells, cellular organelles and extracellular fluid ranging from mm to pm concentrations.^[46]^ Intracellular concentrations of Zn^2+^ are estimated to be in the range of 10–100 μm although most Zn^2+^ is bound in biomolecular complexes. An increase in Zn^2+^ concentration was previously observed in cells under oxidative stress that was mainly attributed to the release of protein‐bound zinc into the cytosol.^[45]^ It is important to note that XRF measures the overall change in Zn concentration rather than the change in unbound versus bound Zn^2+^ concentration. The measured threefold increase in Zn^2+^ concentration in Htt‐ex1‐expressing cells suggests that cells enrich Zn^2+^ from the extracellular fluid using ZIP transporters under these conditions. A possible reason for the increased intracellular Zn^2+^ demand could be the enhanced involvement of Zn‐containing enzymes in the stress response to overexpression of the Htt‐ex1 proteins.^[47]^ Since the PIC‐treated cells show a similar increase in Zn^2+^ concentration, we conclude that the increase cannot be attributed specifically to a Htt‐ex1 misfolding or aggregation.

The concentration of K^+^ in untreated cells is expected to be approximately 100 mm, with values up to 200 mm reported in literature,[^48^, ^49^] depending on cellular conditions such as the cell generation.^[50]^ We measured a K area concentration of 2.53 ng mm^−2^ for untreated cells (Table 1). To compare these values, an average cell thickness of 2.4 μm was estimated by comparing the Si signal at the position of the cells to a position adjacent to the cells. Thus, a concentration of 27 mm could be calculated, which is in good agreement with the values reported in literature. A 17‐ and a 12‐fold increase in K^+^ area concentration was observed for the Htt‐ex1‐transfected cells. A similar effect was observed for the treatment of primary murine astrocytes^[5]^ with Aβ aggregates where a 1.5‐fold increase in intracellular K^+^ was found. Also, increased Na^+^ and K^+^ levels, up to +26 % for Na^+^ and +15 % for K^+^ compared to controls, were found in the brain tissue of Alzheimer's disease patients.[^3^, ^5^] The increase could be attributed to a stress‐induced, ROS‐related damage of K^+^ channels.[^7^, ^51^] The large variance of the K^+^ levels in the Q72 could point towards an even further progressed damage not only of the K^+^ channels, but of the integrity of further ion channels or even the entire cell membrane in a subpopulation of the cells. The PIC‐treated cells showed a similar increase in K^+^ levels (17×), confirming that the effect is related to cellular stress induced by aggregation and not a Htt‐ex1‐specific effect. The small variance in the K^+^ levels throughout the PIC‐treated cellular population points to a highly similar response of all cells and intact cellular integrity. In these cells, apoptotic processes would not explain the K^+^ increase as they commonly lead to an efflux of K^+^ and a decrease of intracellular K^+^, which we did – even after 60 min – not observe.

## Conclusion

We applied μ‐XRF imaging with synchrotron radiation to analyze the elemental distribution in huntingtin‐expressing (Htt‐ex1) cells under the presence of different intracellular aggregates. The change in P, S, K, and Zn area concentration upon Htt‐ex1 and PIC aggregation and the visible light fluorescence microscopy images were measured with 400 nm spatial resolution in order to be able to discuss the data correlatively with optical microscopy and verify the successful transfection of individual cells. In contrast to previous studies considering the Aβ, we found a homogenous partitioning of the elements between cytoplasm and aggregates in Htt‐ex1‐ and PIC‐containing cells. We measured cell‐averaged increases of elemental concentrations upon aggregate formation compared to untreated controls, most significantly for K and for Zn. We attribute this effect to a cellular stress response that is non‐specific to the amylogenic protein Htt‐ex1. We corroborated this hypothesis by investigating the chemically distinct aggregates of PIC in cells. For future studies, we intend to use the technique to investigate ion homeostasis in neuropathological processes on the multi‐cellular to organism level.

## Experimental Section

### Cell Culture Preparation

HeLa cells were cultured in DMEM (Dulbecco's Modified Eagle's Medium, Gibco, Carlsbad, USA) supplemented with 10 % fetal bovine serum (Sigma Aldrich Chemie GmbH, Steinheim am Albuch, Germany) and 1 % penicillin‐streptomycin (Gibco, Carlsbad, USA) at 37 °C and humidified atmosphere with 10 % CO_2_ using T‐25 cell culture flasks (Sarstedt AG & Co, Nümbrecht, Germany). At high confluency, cells were transfected with 2 μg of DNA of IRES constructs of Htt‐ex1 polyQ n‐mCerulean and Htt ex1 polyQ n‐mYFP (1) with different polyglutamine lengths (n=Q25, Q72).^[35]^ Lipofectamine 2000 (Life Technologies GmbH, Germany) was used as transfection reagent. Successful transfection of the cells under investigation was confirmed by fluorescence microscopy. 7,500–10,000 cells were split and seeded onto silicon nitride membranes (1.5 mm×1.5 mm, Silson Ltd., Blisworth, England) and incubated in culture medium for another 12–16 h.

### Microscopy and Cryo Fixation

The adhered cells expressing Htt‐ex1 were imaged using light microscopy (Nikon Ti−E), during which the cells were placed in a PBS (Gibco, Inchinnan, England, Cat Nr.: 18912–014) solution. Utilizing a 20× objective, both, widefield phase contrast and fluorescence microscopy images were recorded. For excitation, an LED (385 nm wavelength) was used, while the light emitted from the sample was detected at 478 nm. HeLa cells treated with 50 μm PIC in Leibovitz's solution (Sigma Aldrich Chemie GmbH, Steinheim am Albuch, Germany) were imaged using confocal microscopy (Olympus IX83). For excitation of mYFP, a 532 nm Laser was used with a 60× oil objective. The emitted light was detected at 575 nm±10 nm. Subsequently, cells on their support membranes were plunge‐frozen in a liquid ethane/propane mixture and stored in liquid nitrogen until the XRF measurements.

### Nanobeam X‐ray Fluorescence Analysis

μ‐XRF measurements for the Htt‐transfected cells as well as for the HeLa cells treated with PIC were carried out at the microprobe‐branch of the Hard X‐ray Micro/Nano‐Probe Beamline P06, DESY (Hamburg).^[36]^ For both experiments, a cryogenic vacuum chamber[^29^, ^32^] was used, enabling measurements in vacuum at a base pressure between 10^−6^ mbar to 10^−8^ mbar at a typical sample temperature of 120 K. The synchrotron beam was focused using KB mirrors.^[37]^ The entrance window into the cryogenic vacuum chamber was made of black Kapton with a thickness of 75 μm (Dupont, Hertfordshire, England). The fluorescence signal was detected by a “Rococo 2” detector (PN detectors, München, Germany), which was positioned in a backscattering geometry upstream of the sample. It uses four monolithically integrated SDD sensor elements (15 mm^2^ each) in an annular cloverleaf shape centered around a hole with a 1.8 mm diameter to allow transmission of the synchrotron beam. This enables the use of the Rococo 2 in close proximity to flat samples at a high acceptance angle of up to 1.1 sr. The 2D area scans were measured in continuous scan mode, in which the sample stages were continuously moved in the horizontal direction, and at each scan end moved incrementally in the vertical direction. During measurements, the X‐ray fluorescence data was collected for a pre‐defined time over a pre‐defined distance using pulse processors from the Xspress line (Quantum Detectors, Oxford). The absolute position of the stages was determined using encoders. The intensity of the excitation X‐ray beam was monitored using a photodiode (I1) and an ionization chamber (I0). For the 2D scans, the spectra of three of the four active elements of the Rococo 2 detector were summed up and normalized to the intensity of the excitation beam, as one of the readout channels was used for diagnostic purposes. The experimental parameters deviated slightly for the Htt‐transfected cells on the one hand, and the HeLa cells treated with PIC as well as the untreated sample P1_Crtl on the other hand. For the Htt‐transfected cells (Table 1), the energy of the synchrotron beam was 12 keV and the focus on the sample was 300 nm×300 nm. The detector was positioned 3 mm upstream of the sample (solid angle≈1.0 sr). The X‐ray fluorescence data was collected using an Xspress 3 pulse processor. For the HeLa cells treated with PIC (Table 2) as well as the untreated sample P1_Crtl, the μ‐XRF measurements and data analysis was different in the following details: The energy of the synchrotron beam was 17 keV and the focus on the sample 440 nm×500 nm. In addition, the detector was positioned 4 mm upstream of the sample (solid angle≈0.9 sr). For analysis of the emitted X‐ray fluorescence photons, two Xspress 3 Mini pulse processors were used.

### Analysis of the X‐ray Fluorescence Data

Due to the open loop operation, the 2D scans with continuous sample axis motion were not strictly spaced on an equidistant grid and were interpolated on a Cartesian grid using the recorded encoder positions. All spectra were analyzed by peak deconvolution and fitting using the fast XRF stacking function of the software PyMCA (European Synchrotron Radiation Facility, ESRF).^[38]^ Area concentrations (areal mass) of the measured elements were obtained by calibrating the XRF experiment with a thin film XRF reference sample “RF17‐200‐S4218‐41“ (AXO Dresden, Germany). The thickness of the amorphous ice of the rapid plunge‐frozen HeLa cells was determined by comparing the intensity of the mean Si K signal I(K_Si,ice_) at the position of the cells to the Si K signal obtained from a spectrum of an empty silicon nitride membrane (I(K_Si,membrane_)) following recently published protocols.^[29]^ As transmission of the Si K signal with a photon energy of 1.74 keV, an attenuation length in water of λ=11.6 μm was used.[^29^, ^39^] For the investigation of aggregate formation in Htt‐ex1‐transfected cells, 7 Q25‐transfected (25267 XRF spectra) and 18 Q72‐transfected cells (101483 spectra) were analyzed. For the local analysis of element levels in Htt‐ex1‐expressing cells, light fluorescence microscopy was used prior to the μ‐XRF measurements to localize the fluorescently labeled inclusion bodies. The light microscopy images were correlated with the corresponding XRF maps to determine the element area concentrations in inclusion bodies (IBs). The resulting area concentrations were compared with corresponding concentrations from areas adjacent to (IBs). For PIC‐treated cells, the element levels in 45 cells after 10 min incubation in the presence of PIC (120000 spectra), and 52 cells after 60 min incubation in the presence of PIC (138297 spectra) were measured. Examples of XRF spectra are given in Figure S1. The identical XRF dataset of 17 wild type cells (44910 spectra) has been used as an untreated control for comparison to Htt‐ex1‐transfected and PIC‐treated cells.

## Conflict of interest

The authors declare no conflict of interest.

1

## Supporting information

As a service to our authors and readers, this journal provides supporting information supplied by the authors. Such materials are peer reviewed and may be re‐organized for online delivery, but are not copy‐edited or typeset. Technical support issues arising from supporting information (other than missing files) should be addressed to the authors.

Supporting Information

## Data Availability

The data that support the findings of this study are available from the corresponding author upon reasonable request.
