# Supplementary material for: Spatial Distribution of Intracellular Ion Concentrations in Aggregate‐Forming HeLa Cells Analyzed by μ‐XRF Imaging
Source: ChemistryOpen. 2022 Apr 1;11(4):e202200024. doi: 10.1002/open.202200024 (PMC8973254; doi:10.1002/open.202200024)
Supplement: Supplementary file 1 — Supporting Information [file OPEN-11-e202200024-s001.pdf]

ChemistryOpen

Supporting Information

Spatial Distribution of Intracellular Ion Concentrations in Aggregate-Forming HeLa Cells Analyzed by μ-XRF Imaging

Andreas Gräfenstein, Christoph Rumancev, Roland Pollak, Benjamin Hämisch, Vanessa Galbierz, Walter H. Schroeder, Jan Garrevoet, Gerald Falkenberg, Tobias Vöpel, Klaus Huber, Simon Ebbinghaus,* and Axel Rosenhahn*

Table S1-S3 provide the statistical ANOVA tests to reveal the statistical significance of observed differences.

Table S1:ANOVA of the area concentrations (areal mass) in untreated and huntingtin-transfected cells. Average single cell concentrations are given in Table 1 of the main manuscript. Values below 0.05 indicate statistical significances in element concentrations.

	P	S	K	Zn
**significance 0.05**	**p-value**	**p-value**	**p-value**	**p-value**
**Untreated – Q72**	2.71714E-8	1.15415E-9	1.73986E-4	0
**Untreated – Q25**	0.09914	1.52872E-5	1.00127E-4	8.36866E-7
**Q72 – Q25**	0.00641	0.95778	0.40338	0.9968

Table S2: ANOVA of the area concentrations (areal mass) in areas rich and poor in cellular inclusion bodies containing Htt-ex1 aggregates in the same transfected cells (s. Figure 3 & S2). Values below 0.05 indicate statistical significances in element concentrations.

	P	S	K	Zn
**significance 0.05**	**p-value**	**p-value**	**p-value**	**p-value**
**Q25/1 (186)**	1.92004E-5	3.1366E-6	0	1.60113E-6
**Q25/2 (189)**	1.15555E-5	1.04246E-5	0	0.20157
**Q72 / 1 (106 #1)**	0.98169	2.18666E-7	3.0624E-4	2.44375E-8
**Q72 / 2 (134 #2)**	0.01416	3.9562E-7	0.80802	2.5977E-6
**Q72 / 3 (136 #4)**	0.99896	0.10699	0.96139	0.66702
**Q72 / 4 (204 #1)**	0.9876	0.07383	0.00937	0.15435
**Q72 / 5 (204 #2)**	0.5662	0.05009	0.99799	0.00114

Table S3: ANOVA of the area concentrations (areal mass) in untreated and PIC treated cells. Average single cell concentrations are given in Table 2 of the main manuscript. Values below 0.05 indicate statistical significances in element concentrations.

	P	S	K	Zn
**significance 0.05**	**p-value**	**p-value**	**p-value**	**p-value**
**Untreated - 10 min**	4.72774E-9	2.2103E-12	1.40683E-20	6.49951E-18
**Untreated - 60 min**	1.12426E-5	1.24635E-10	4.65319E-19	5.95542E-16
**10 min - 60 min**	1.61296E-5	0.00412	8.99282E-4	1.36385E-4

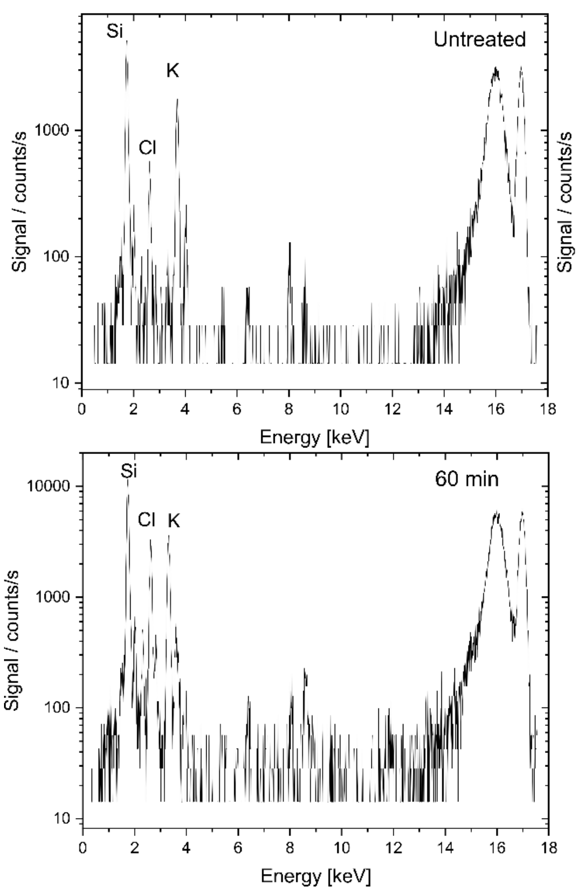

Figure S1: XRF single spectra of untreated cells and cells after different incubation time with PIC.

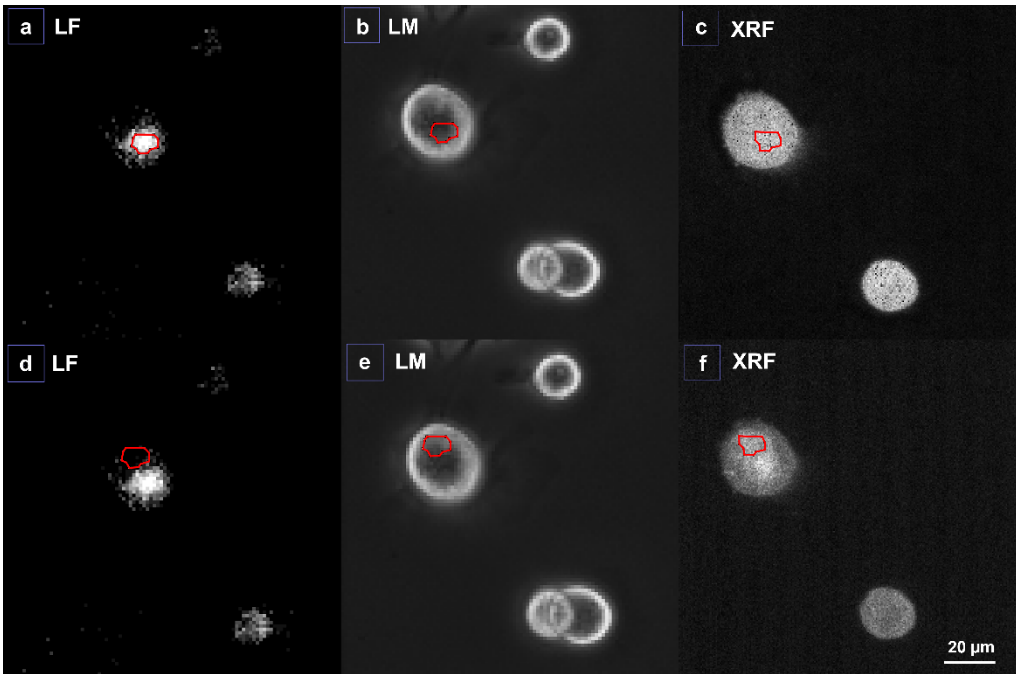

Figure S2: Exemplary selection procedure of regions rich (a-c) and poor (d-f) in cellular inclusion bodies containing Htt-ex1 aggregates for the corresponding concentration analysis within a single cell. The selection area is marked by a red line. First, the rich region was manually selected in the light fluorescence image (a), which is correlated to the light microscopy image (b). Then, a similar selection area within the cell was chosen in the XRF images (c) to calculate the elemental area concentrations within the selected pixels. For the poor regions, the selection area in the light fluorescence image was moved to an area within the cell with no elevated signal (d) and then the procedure was repeated as described. XRF images of S and Zn are shown in c) and f), respectively.

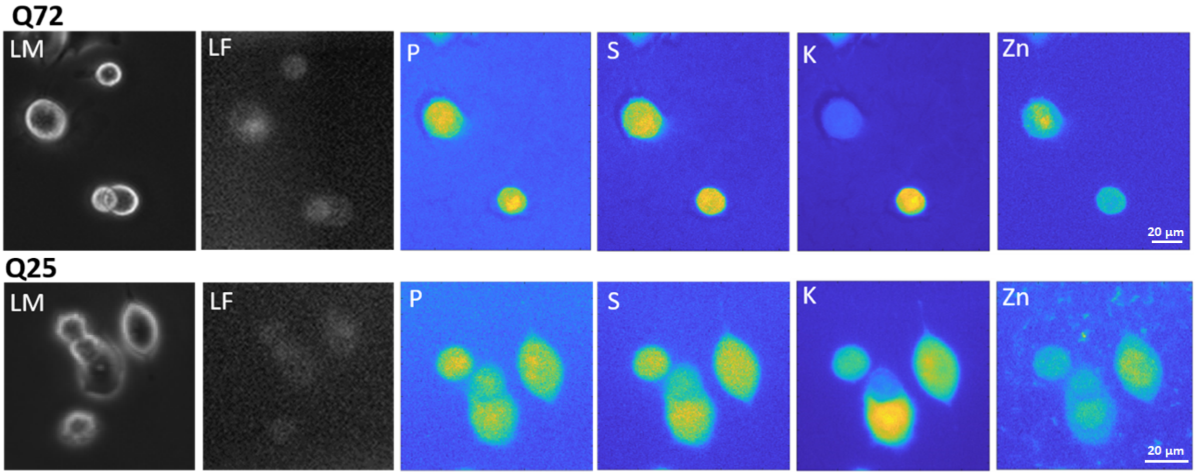

Figure S3: Exemplary phase contrast microscopy (LM) and visible-light fluorescence (LF) microscopy as well as X-ray fluorescence images of individual Htt-transfected HeLa cells with different polyglutamine lengths (Q72 & Q25). An inhomogeneous concentration distribution can be seen for all shown elements within the cells but is most pronounced for Zn, which exhibits an increased concentration in and around the nucleus. The Q72-transfected cell in the upper left in the top row shows a strongly reduced K concentration compared to the cell in the bottom right. This K depletion could be observed for 10 out of the 18 Q72- and 2 out of the 7 Q25-transfected cells measured (s. Figure 2).
